# Closing the Mind's Eye: Incoming Luminance Signals Disrupt Visual Imagery

**DOI:** 10.1371/journal.pone.0015217

**Published:** 2010-12-20

**Authors:** Rachel Sherwood, Joel Pearson

**Affiliations:** The School of Psychology, The University of New South Wales, Sydney, Australia; Indiana University, United States of America

## Abstract

Mental imagery has been associated with many cognitive functions, both high and low-level. Despite recent scientific advances, the contextual and environmental conditions that most affect the mechanisms of visual imagery remain unclear. It has been previously shown that the greater the level of background luminance the weaker the effect of imagery on subsequent perception. However, in these experiments it was unclear whether the luminance was affecting imagery generation or storage of a memory trace. Here, we report that background luminance can attenuate both mental imagery generation and imagery storage during an unrelated cognitive task. However, imagery generation was more sensitive to the degree of luminance. In addition, we show that these findings were not due to differential dark adaptation. These results suggest that afferent visual signals can interfere with both the formation and priming-memory effects associated with visual imagery. It follows that background luminance may be a valuable tool for investigating imagery and its role in various cognitive and sensory processes.

## Introduction

It has been proposed that mental imagery is akin to perception in the absence of external stimulation, a product of sensory information retrieved from memory [1]. It is often referred to as seeing with the mind's eye, hearing with the mind's ear, or feeling with the mind's skin [2]. We use imagery on a daily basis during high-level cognitive tasks such as navigation, spatial planning, or even to remember a friend's face [1], [3]. It has also been proposed that mental imagery contributes to memory storage and retrieval [1]. Mental imagery has even been used as an effective tool in cognitive therapy and for improving motor abilities through mental practice [4], [5], [6].

An ongoing debate in the literature is whether mental imagery involves pictorial low-level mechanisms, or whether it is symbolic in a similar vein to language, and thus a more high-level mechanism [1], [7]. Evidence for it being pictorial in nature comes from research showing that mental imagery is processed retinotopically [8], [9]. These researchers found that two-thirds of brain activity observed during mental imagery was also activated during visual perception. Furthermore fMRI studies of visual imagery have shown that, as with normal vision, the brain areas activated depend on the type of object being visualised [8], [10]. In addition, behavioural methods have been used to investigate the nature of imagery [11], [12], [13], [14], and produced strong evidence for its similarity with visual perception.

Pylyshyn (1973) on the other hand, suggested that imagery is not pictorial in nature but rather represented and stored by propositions in the same way as semantic information is represented by its relationship with other concepts. In this scenario the observed similarities between imagery and perception might simply be due to our ability to compose imagery in a way that resembles perception, not because they are inherently similar processes [7], [15].

Strong evidence that imagery involves mechanisms in early visual processing comes from an empirical method developed by Pearson, Clifford and Tong (2008) in which imagery was used to bias subsequent binocular rivalry. Binocular rivalry refers to the fluctuations in visual awareness that result from displaying two different patterns, one to each eye. When participants were cued to imagine one of the rivalry patterns, imagery tended to prime subsequent perception of rivalry. The pattern a subject saw during the rivlary presentation tended to be the pattern they had imagined a few seconds earlier. In addition, it was shown that this priming was orientation specific, that is, for this bias to occur, imagery had to be at the same angular orientation as the rivalry patterns. This work sugests that visual imagery involves low-level mechanisms that overlap with visual perception [16].

Pearson et al. (2008) also showed that the higher the level of background luminance during the binocular rivalry task, the weaker the effect of imagery on subsequent perception. This suggests that the general level of background luminance can attenuate imagery's effect on subsequent perception. When using binocular rivalry to investigate imagery, to avoid any confounding effects of visual attention on perception, the authors always separated imagery and perception in time. It is well known that visual attention can change the perceived characteristics of a visual stimulus [17]. Separating imagery and perception in time introduces a memory trace between imagery generation and the binocular rivalry test stimulus. Hence, in the above study it was unclear if the luminance of the background was attenuating imagery generation, the memory trace, or both.

Little is known about the nature of the memory trace formed between imagery and the rivalry task. It is known to last several seconds [12], therefore outside the realms of iconic memory, which only lasts for up to around 500 ms [18], [19]. Additionally, the memory for imagery is automatic unlike visual spatial working memory, which typically requires active maintenance [20], [21]. This memory seems to be more akin to the memory during perceptual priming as it is specific to the features and location of the stimulus being imagined [12], [22], [23].

In the current study we report that background luminance can attenuate both mental imagery generation and storage of the imagery trace. However, imagery generation seems to be more sensitive to luminance, as lower levels of luminance had greater effects on generation compared to the memory trace. In addition, when luminance was presented between experimental trials outside of imagery generation *and* storage, these effects were not observed.

## Methods

### Participants

Thirteen undergraduate psychology students completed all conditions of the first two experiments. An additional nine participants were screened who did not meet a criterion of imagery priming above 50% ( = >51% bias as measured by the rivalry task in the no luminance condition). The criterion was implemented in order to exclude participants with very weak imagery, indicated by a lack of priming in the binocular rivalry task. For this study we were interested in how background luminance would attenuate processes associated with imagery. Hence, we required subjects who displayed a moderate or high level of imagery in the dark no luminance condition. Fourteen participants took part in the third experiment, with four who did not meet the above criterion. Subjects gave informed written consent before participating in the experiment, and received payment for participation in the form of course credit. The local Human Research Ethics Advisory Panel (Psychology) at The University of New South Wales approved this study.

### Pre-experiment Tests

The stimuli were red and green sinusoid luminance modulated oriented gratings. The contrast of the stimulus was applied to a Gaussian-windowed mean luminance profile (spatial frequency 1.6 cycles/°, Gaussian  = 2.6°), which had a mean luminance of 4 cd/m^2^. These oriented patterns had an internal contrast of ∼60% in relation to the average luminance of the stimulus only, as the surrounding background was black. The red grating pattern comprised of a CIE of x = .277, y = 0.613 with a horizontal orientation, presented to the left eye. The green vertical pattern (CIE of x = 0.601, y = 0.368) was presented to the right eye. Both patterns were presented in an annulus around a bullseye fixation spot 0.5° in diameter.

In order to minimize potential eye bias, an initial task was completed to determine the correct balance of stimulus contrasts between the two rivalry patterns. The contrast of the gratings had to be adjusted for each participant in order to account for individual differences in eye dominance and bias. In order to determine these contrasts, the same procedure was used as in previous studies see [12]. This procedure involves exposing participants to an intervening perceptual stimulus (one of the rivalry patterns) that acted to adapt or fatigue the neurons representing a particular pattern. This procedure has been previously used successfully to attain equivalent functional strengths between the two rivalry patterns.

This eye bias procedure involved each subject being presented with the rivalry patterns, in isolation (just one pattern) and subsequently together undergoing rivalry. Each of the isolated presentations lasted 4 seconds. Not only did this procedure allow us to equalize the pattern strengths, it also provided an opportunity for subjects to familiarize themselves with each of the patterns they would subsequently be required to imagine. In addition, before experimental trials were allowed to continue subjects were shown hard copy color printouts of each grating pattern and verbally asked if they felt familiar enough with each pattern to perform mental imagery of that pattern. Participants were asked to imagine the patterns as best they could and if possible to imagine the color, spatial frequency, size and location in visual space of the pattern.

All conditions of the experiment were conducted in a darkened room (0 cd/m^2^ reflectance off a white surface) with black walls, on a calibrated (19″ Philip Brilliance 109P4) monitor with a resolution of 1280×960 and a refresh rate of 75 Hz. The experiment was run in MatLab and the Psychophysics toolbox [24], [25] on a Mac Mini computer.

### Main Experiment

**Figure 1:**
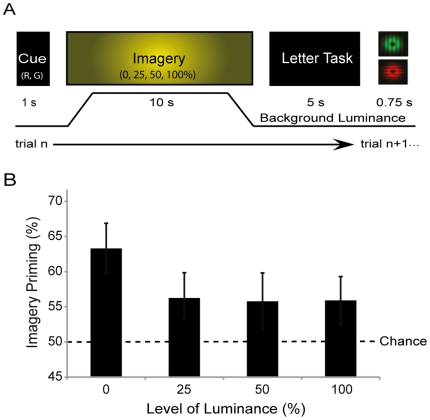
Experiment one: The effect of luminance on imagery generation. (**A**) Time-line showing the different phases of a trial and the rivalry stimuli. First the letter cue, presented on a black background indicates the pattern to imagine. The background during the imagery task is illuminated, at 0, 25, 50 or 100% of the mean luminance of the rivalry patterns, followed by a 5 second long letter discrimination task and then the rivalry display. (**B**) The percentage of trials in which the dominant rivalry pattern matched the imagery cue, grouped by luminance of the background. Note that priming of 50% indicates chance. The error bars show the standard error of the mean (SEM). N = 13.

In the first experiment participants were randomly cued to imagine one of the binocular rivalry patterns for 10 seconds. “R” was the cue for the red horizontal grating and “G” for the green vertical grating (see figure 1a). During the imagery period one of four different background luminance levels was presented. These varied from 0% (black screen), 25%, 50% and 100% of the mean luminance and colour of the two rivalry patterns (4 cd/m^2^). These conditions were run in blocked sets of experimental trials. The background luminance ramped on and off smoothly over 500 ms to avoid visual transients.

Following imagery generation, participants completed a rapid serial visual presentation task (RSVP) in which random letters were presented on the screen for a five second period, displayed for 300 ms each. Behind each letter was a small Gaussian patch of luminance matching the background in the 100% background luminance condition, this remained the same in all conditions. This was to maintain consistency in the letter task throughout all conditions. Participants were required to press the “C” or “V” key as soon as either letter was presented on the screen. The RSVP task was included as a distracter task to ensure that participants stopped imagining the stimulus at the required time. This task also ensured that any influence on rivalry was not due to an attentional effect as the participants had to apply their attention to the RSVP task. This was lastly followed by a binocular rivalry display for 0.75 seconds. Participants indicated the dominant pattern using 3 different keys, either “1” green, “2” for a mixed percept or “3” for the red pattern. The trials in which participants reported seeing a mixed percept were discarded from the analysis. If a subject reported a mixed percept on more than 20% of trials they were dropped from further analysis.

There were at least 60 trials for each level of background luminance for each subject. Each block of 60 trials lasted for ∼20 minutes. In addition, ten practice trials were completed before testing began.

**Figure 2:**
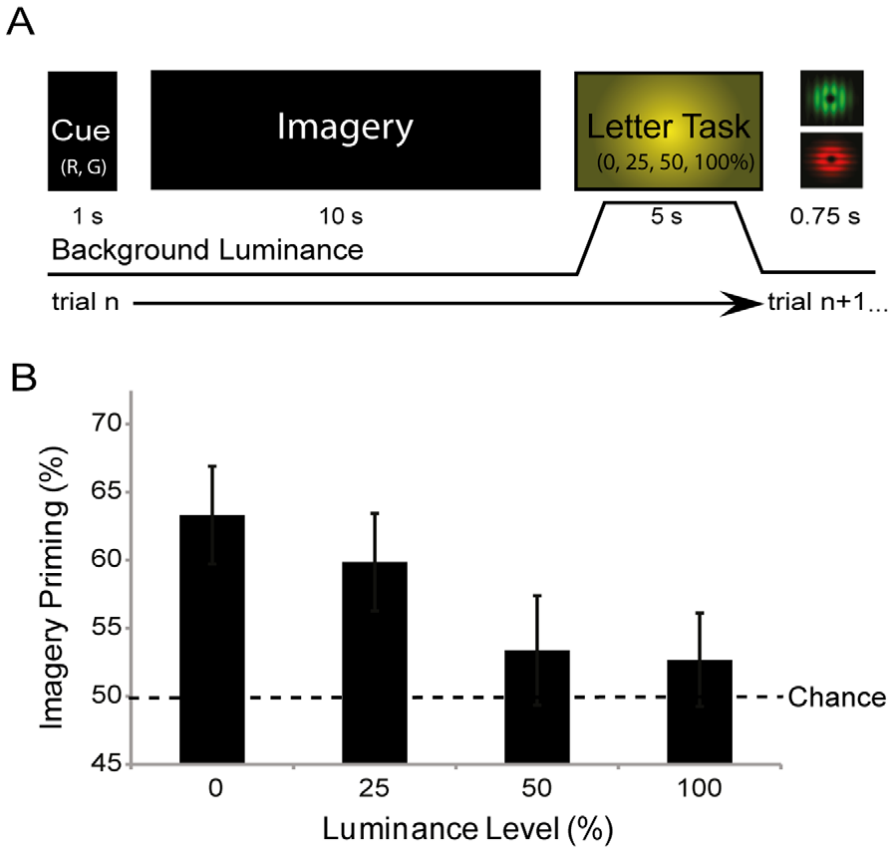
Experiment two: The effect of luminance on imagery storage. (**A**) The time-line, here was the same as that is experiment one, with the exception that the different levels of background luminance were shown during imagery storage (letter task). (**B**) The percentage of trials in which participants were primed by imagery grouped by luminance of the background during the letter task/storage period. The error bars show the standard error of the mean (SEM). N = 13.

**Figure 3:**
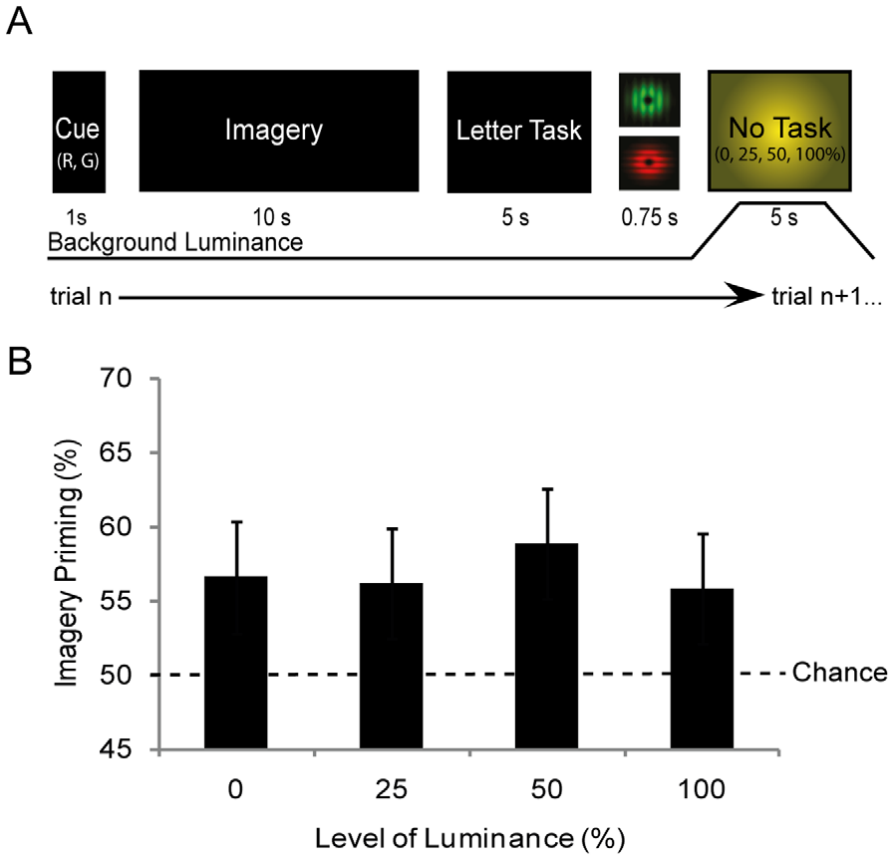
Experiment three: The effect of luminance between experimental trials. (**A**) Experimental time-line, here was the same as that in experiment one and two, with the exception that the different levels of background luminance were shown between each experimental trial. (**B**) The percentage of trials in which participants were primed by imagery grouped by luminance of the background between each trial. There was no significant difference between imagery priming for the different luminance backgrounds. The error bars show the standard error of the mean (SEM). N = 14.

The second experiment was otherwise the same, however, the luminance backgrounds were presented during the RSVP letter task, rather than during imagery generation (see figure 2a). A final experiment was conducted with a separate group of participants in order to test for any influence of dark adaptation, as the change in the sensitivity due to long periods in the dark could potentially influenced the interaction between imagery and binocular rivalry perception. In this experiment the four levels of background luminance were presented between each experimental trial for 5 seconds (see figure 3a). Furthermore, the order of conditions and luminance levels was pseudo-randomized in all experiments.

## Results

### Experiment One: Imagery Generation

Experiment 1, examined the effect of different levels of luminance during the imagery generation period. Figure 1b shows the data from experiment 1. In the no luminance condition (leftmost column) imagery primed the dominant percept in the subsequent rivalry presentation in 63% of trials. Whereas in the 25, 50 and 100% luminance conditions imagery only primed rivalry dominance ∼55%, showing an overall effect of luminance (F_(1,12)_ = 7.2, *p* = 0.001). The 25, 50 and 100% conditions did not significantly differ from the chance score of 50%, (lowest *p* = 0.178), but were all significantly different from the dark 0% condition (highest *p* = 0.01).

### Experiment Two: Imagery Storage

In experiment 2 we examined how imagery's ability to prime rivalry was influenced by different levels of background luminance during the storage period in which subjects performed the letter detection task. The results of experiment 2 are shown in Figure 2b. Imagery priming decreased as the intensity of the background luminance increased. In the 25% luminance condition imagery primed rivalry dominance in 60% of trials, while in the 50% and 100% luminance condition imagery primed rivalry around 53% of trials. There was a significant main effect of luminance during the storage period, on rivalry priming (F_(1,12)_ = 6.698 *p* = 0.001).

Unlike experiment one, here the 25% luminance condition was not significantly different from the dark 0% condition (t = −1.358, *p* = 0.199), and was significantly different from chance 50% (t = 2.753, *p* = 0.017). Suggesting the memory trace of imagery is less sensitive to background luminance as compared to the imagery generation process.

### Experiment Three: Dark Adaptation

The current study was conducted in the dark the majority of time and it is known that dark adaptation occurs after a period of time in darkness [26], [27]. In experiment one and two, participants were exposed to luminance during different periods of an experimental trial. Luminance during both the generation and storage periods affected imagery's ability to prime subsequent rivalry, albeit somewhat differently. To investigate the possibility that exposure to luminance at any time period in close temporal proximity to imagery might attenuate its effects on subsequent vision, we ran an experiment in which we manipulated the luminance between experimental trials. The luminance between each experimental trial could be one of either 0%, 25%, 50% or 100% background luminance.

Figure 3b shows the data form this experiment. There was no significant effect of luminance across the different conditions (F_(1,13)_ = .761, *p* = .523). This data suggests that the effect of luminance in the previous experiments was immediate and local in time.

### RSVP Letter Task

There was no significant correlation between the performance on the RSVP letter task and imagery priming (R = 0.264, *p* = 0.26). In addition, there was no significant effect of luminance on the RSVP task scores in either experiment one (F = 1.28, *p* = .3) or two (F = .820, *p* = .5).

## Discussion

The results of the current study indicate that both imagery generation and imagery storage can be attenuated by background luminance. This finding is consistent with the results of Pearson et al. (2008), who showed that forming a mental image and storing it in the presence of an illuminated screen results in weaker effects on subsequent perception. Furthermore, imagery generation seems to be more sensitive to luminance than imagery storage, as even the lowest amount of background luminance during imagery generation caused a significant decrease in imagery priming. The lowest level of background luminance, on the other hand, did not significantly disrupt the imagery memory trace. In addition, these results do not appear to be due to the general presence of luminance outside the trial sequence.

Various explanations could be proposed to account for the effect of luminance on imagery. As the luminance ramped on slowly and remained constant until the end of the designated time period, it seems unlikely that the presence of luminance simply distracted subjects from performing the imagery task or led to increased perceptual load [28]. In addition, the presence of luminance had no significant effect on performance in the letter detection task.

Both afferent visual signals originating from the illuminated background and the neural response related to imagery can involve activity in early visual areas [9], [12], [29], [30]. One proposition is that activity in these early visual areas might have plateaued due to the incoming luminance based signals. If this were the case, the addition of any top-down neural activity due to imagery may have a negligible net effect on the absolute level of neural firing eg. a ceiling effect.

Experimental participants here, and in previous studies [12] often commented that forming a mental image somehow felt more difficult in bright illuminated environments. In fact, the absence of incoming visual signals has been associated with hyper-imagery ability [31]. Interestingly, dynamic visual noise has been shown to influence spatial imagery tasks [32] and also visual spatial working memory tasks [33]. Recent work has demonstrated that the presence of task irrelevant visual displays can even interfere with long-term memory retrieval [34].

The affects of dynamic visual noise have been used to illustrate commonalities between visual spatial working memory systems and visual imagery [32], [33]. In fact, the degree of vividness of mental images has been linked to the specific architecture of the visual spatial working memory system [35], [36], [37]. However, it remains to be seen if the degree of background luminance can be utilised to untangle the seemingly intricate relationship between visual imagery and visual spatial components of working memory. It is worth noting that in previous work [12], the degree of background luminance while attenuating the effects of visual imagery did not systematically alter the affects of visual attention.

The results of the present study may support the pictorial nature of imagery. Luminance was able to disrupt imagery generation, and since luminance and imagery both activate early visual areas, it could be suggested that this interaction is occurring in early visual areas known to process visual stimuli and imagery in a pictorial/retinotopic manner [9], [30], [38].

Given that imagery generation is more sensitive than storage to luminance, it is possible that the cognitive process implicated in storage is actually different to that during generation. Therefore, imagery may engage two separate mechanisms, one for imagery generation and another for imagery storage. The nature of this imagery memory trace is yet to be fully explored. However, one possibility is that the memory involved in imagery is similar to perceptual memory, particularly perceptual priming [23]. In Pearson et al (2008), visual imagery was shown to have a similar influence on binocular rivalry as weak visual stimulation, suggesting that the memory traces involved in imagery and perception might be a part of the same memory system. Evidence suggests that this type of perceptual memory is most probably stored in early stages of processing in the visual cortex [23] and therefore the memory trace for visual imagery may also be stored within similar areas of the visual cortex.

This study shows that visual imagery can be attenuated by afferent visual signals and therefore imagery can be most effectively utilized and examined in dark environments. This has implications for future studies that aim at investigating the nature of imagery and its roll in cognitive functioning. Furthermore, the current data suggest the existence of two partially separable mechanisms associated with imagery, one for generation and one for storage. This memory mechanism for imagery resembles the processes underlying perceptual memory. However, the full extent of any mechanistic overlap is an interesting topic for future studies. We hope that the manipulation of afferent visual signals could be a valuable investigative tool giving traction to future studies of mental imagery.
